# Cardiovascular Risk Stratification and Surveillance in Cardio-Oncology: Mechanistic Foundations and Future Directions

**DOI:** 10.3390/biomedicines14081771

**Published:** 2026-08-06

**Authors:** Aryan Gajjar, Syed Asfand Yar Shah, Amani Gajjar, Krishna C. Allam, Bryce Beech, Andrew Pfaff, Claiborne Brochu, Sourbha Dani, Sanju Ganatra

**Affiliations:** 1 Department of Internal Medicine, Division of Cardiology, David Geffen School of Medicine at UCLA, Los Angeles, CA 90095, USA; 2 Department of Cardiology, Mayo Clinic Institute, Rochester, MN 55905, USA; 3 Northern Light Eastern Maine Medical Center, Bangor, ME 04401, USA; 4 Department of Internal Medicine, Tulane School of Medicine, New Orleans, LA 70112, USA; 5 Department of Medicine, George Washington School of Medicine and Health Sciences, Washington, DC 20052, USA; 6 Department of Biology and Biomedical Sciences, Salve Regina University, Newport, RI 02840, USA; claiborne.brochu@salve.edu; 7 Division of Cardiovascular Medicine, Department of Medicine, Lahey Hospital and Medical Center, Burlington, MA 01805, USA

**Keywords:** cardio-oncology, cancer therapy-related cardiotoxicity, cardiovascular surveillance, risk stratification, precision medicine

## Abstract

Cardio-oncology has emerged as a critical discipline in modern medicine due to the growing population of cancer survivors and the increasing recognition of cardiovascular disease as a major cause of morbidity and mortality in this population. While there have been significant advances in chemotherapy such as advances in chemotherapy, targeted therapies, immunotherapies, and radiation therapy, these treatments are linked to a wide range of cardiovascular toxicities such as myocarditis, arrhythmias, and progressive fibrotic remodeling. These toxicities tend to be caused by interrelated and multifactorial cellular processes including endothelial damage, immunological dysregulation and mitochondrial dysfunction. Therefore, early diagnosis of subclinical harm using multimodal surveillance strategies integrating clinical risk assessment, specialized clinical imaging, and circulating biomarkers has replaced reactive care of overt cardiotoxicity in modern cardio-oncology. Echocardiographic techniques such as longitudinal strain, with the help of biomarker-guided surveillance of natriuretic peptides and cardiac troponins, have made the early detection of cardiac dysfunction more effective. However, the current prediction risk models are still constrained and limited by their dependence on static clinical variables and their partial integration of biological mechanisms. This review examines the current methods for risk stratification and surveillance throughout the cancer care continuum, including baseline assessment, monitoring during active therapy, and long-term survivorship surveillance. Future approaches for tailored cardiovascular care are also highlighted, including new advances in precision cardio-oncology such as multi-omics profiling, molecular biomarkers, artificial intelligence, and mechanism-guided preventative strategies. Therefore, advancing biologically informed and risk-adapted monitoring frameworks may enhance early diagnosis, maximize cardioprotective measures, and lower long-term cardiovascular consequences.

## 1. Introduction

Cardio-oncology has established itself as an important discipline in health care, due to the rapidly expanding population of cancer survivors. Many of the surviving patients experience cardiovascular disease, which is the leading cause of morbidity and mortality in this group. Advances in cancer therapeutics have significantly increased the survival rate, with over 17 million cancer survivors currently living in the United States, a number projected to exceed 22 million by 2030 [1]. However, these gains have been accompanied by an increasing risk of cardiovascular disease, which now leads the non-malignant cause of death among cancer survivors [2]. In certain populations, including breast cancer and Hodgkin lymphoma survivors, cardiovascular mortality may even surpass cancer-related mortality over time [3]. Overall, up to 30–40% of long-term cancer survivors develop cardiovascular complications, signifying the importance of cardiovascular risk stratification and surveillance in this population [4].

Cardiotoxicities associated with cancer therapies extend well beyond classic underlying causes such as left ventricular systolic dysfunction. While anthracycline-induced cardiomyopathy is still a well-recognized entity, with clinical heart failure occurring in approximately 5% of patients at standard doses and up to 25–30% at higher cumulative exposures, subclinical myocardial dysfunction may occur in as many as 50% of treated individuals [5,6]. Similarly, HER2-targeted therapies are associated with left ventricular dysfunction in 10–20% of patients [7]. Importantly, newer therapeutic modalities have introduced additional cardiovascular risks, such as immune checkpoint inhibitors, which are associated with myocarditis, although relatively rare (~1%), and carry a mortality rate approaching 40–50% [8]. Radiation therapy also results in long-term cardiovascular risk, with the rate of major coronary events increasing by approximately 7% per Gray (Gy) radiation exposure [9]. This demonstrates how cardiotoxicity is not limited to a single phenotype but instead encompasses a broad spectrum of myocardial, vascular, and electrophysiological complications.

The main challenge in cardio-oncology is the delayed response to the medication that patients face. Up to 98% of cardiotoxicity cases are initially asymptomatic and may not manifest until years or decades after exposure to cancer therapy [10,11]. This is notably evident in childhood cancer survivors, who face a 15-fold increased risk of developing heart failure compared with the general population [12]. These observations emphasize the need to modify risk surveillance approaches that extend well beyond the completion of cancer therapy.

Current surveillance strategies depend on multimodal approaches that incorporate imaging, circulating biomarkers, and clinical risk assessment tools. Echocardiography with global longitudinal strain (GLS) has become known as a sensitive modality for the early detection of subclinical myocardial dysfunction, with a relative reduction in GLS of greater than 15% predicting subsequent declines in ejection fraction [13]. Biomarkers such as cardiac troponins and natriuretic peptides further strengthen early detection, with troponin elevation during chemotherapy conferring approximately a four-fold increased risk of cardiotoxicity [14] and elevated natriuretic peptides linked with future heart failure risk [15]. Even with these advances, existing clinical risk prediction models show only modest performance, with C-statistics frequently ranging from 0.60 to 0.70 and may fail to identify a substantial proportion of patients who ultimately develop cardiotoxicity [16,17].

Findings such as immunological dysregulation, endothelial dysfunction, mitochondrial injury, and maladaptive fibrotic remodeling have increased interest in multi-omics profiling, molecular biomarkers, mechanism-informed risk stratification, and prediction models aided by artificial intelligence. Even though these new methods have shown encouraging outcomes in preliminary studies, the majority are still in the exploratory stage and need prospective validation before being widely used in clinical settings [18].

Numerous recent reviews have looked at particular aspects of cardio-oncology, such as precision medicine techniques, cardiovascular surveillance, or causes of cardiotoxicity. The molecular mechanisms underlying cardiovascular injury and the clinical frameworks utilized for risk classification and surveillance along the whole cancer care continuum are, however, often explored separately. Additionally, relatively few evaluations discriminate between established clinical practice and developing experimental technology while critically assessing the advantages and disadvantages of current surveillance systems. As a result, this review combines modern methods for cardiovascular risk assessment and monitoring with the molecular foundations of cancer therapy-related cardiovascular disease.

In this review, in order to improve precision cardio-oncology through biologically informed and risk-adapted cardiovascular therapy, we evaluate the evidence for novel technologies, critically examine the flaws in existing risk prediction models, and determine future research objectives. In order to support individualized patient care, we also offer a clinically focused framework that connects the mechanistic foundations of cardiovascular toxicity associated with cancer therapy with clinical phenotypes, cardiovascular risk stratification, therapy-specific surveillance throughout the cancer care continuum, and new precision cardio-oncology techniques.

## 2. Mechanistic Foundations of Cancer Therapy-Related Cardiovascular Injury

It is well known that a host of biological pathways normally generate physiologic levels of reactive oxygen species (ROS) and reactive nitrogen species (RNS), including the hydroxyl radical (OH), singlet oxygen (^1^O_2_), hydrogen peroxide (H_2_O_2_), nitric oxide (NO), and nitric dioxide (NO_2_), which are then neutralized by antioxidant enzymes such as catalase (CAT), superoxide dismutase (SOD), and glutathione peroxidase (GPX) [19,20]. These molecules are produced by enzymes in both the cytosol and mitochondria, notably NADPH oxidase (NOX), xanthine oxidase, cyclooxygenase (COX), monoamine oxidase (MAO), and cytochrome P450 (CYP450) [20,21]. In physiological states, these molecules aid in excitation–contraction coupling, maintaining vascular tone, and calcium homeostasis [20]. In pathological states, ROS and RNS can both be produced by and induce the production of themselves from the mitochondria [21]. This, along with mitochondrial DNA damage and mitochondrial permeability transition (MPT), leads to a failure of normal functioning of the mitochondria to generate energy for the cell, which is termed mitochondrial dysfunction [22].

Increased ROS produced by chemotherapeutics or mitochondrial dysfunction can lead to arrhythmias, cardiac dysfunction, fibrosis, induction of cardiomyocyte apoptosis, and hypertrophy, which can manifest as heart failure [21]. Arrhythmias are thought to be induced through oxidative changes to the ryanodine receptor and sarcoplasmic/endoplasmic reticulum Ca^2+^ ATP-ase (SERCA), which can lead to increased cytosolic calcium and cardiac dysfunction [21]. Cardiomyocyte apoptosis can occur through modulation of the intrinsic apoptotic pathway involving the MPT, Bax/Bad translocation to the mitochondrial membrane, and ligand-independent activation of various signaling cascades [21]. Finally, hypertrophy and fibrosis result from the modulation of G-protein-coupled signaling pathways and the activation of TGF-beta, a transcription factor that can activate interstitial fibroblasts into myofibroblasts, respectively [21,23]. These pathways are implicated in the CTX of various chemotherapeutics, as described below.

Anthracyclines (ANTs) are a form of antitumor antibiotics that were first isolated from *Streptomyces peucetius* var *caesius* in the 1960s and entered clinical practice in the 1970s [24]. Doxorubicin (DOX) was one of the first discovered ANTs and remains among the most used chemotherapy agents with activity against many solid and hematologic malignancies, especially when combined with other agents such as 5-fluorouracil (5-FU), cyclophosphamide, and cisplatin [25]. ANTs primarily generate their antitumor activity by (1) generating reactive oxygen species, (2) binding to topoisomerase 2-α (Top-2α), (3) intercalating into DNA, preventing transcription, and (4) altering membrane conductivity and ion transport [25,26]. ANT CTX can be either acute or chronic [25]. Acute ANT CTX is characterized by myocarditis, arrhythmias, and pericarditis, whereas chronic ANT CTX is characterized by progression to HF, sometimes years after treatment [21,27].

Though the production of ROS and RNS by ANTs is pivotal for their antitumor effects, it is also thought to be one of the primary mechanisms by which they cause CTX, especially HF [19,28,29]. One of the first proposed mechanisms by which ANTs can produce ROS is by redox cycling, where the ANT cycles between quinone and semiquinone moieties, producing superoxide (O^2-^) as a byproduct [30]. This can then be converted into H_2_O_2_, which can undergo the Fenton reaction in the presence of iron to generate the highly toxic OH, which oxidizes lipids, damages DNA, and can induce mitochondrial dysfunction [19,28]. This mitochondrial dysfunction is potentiated by the preferential accumulation of ANTs in mitochondria when one of the rings is reduced to an alcohol [28]. The role of iron in ANT cardiotoxicity is well established and may be due to ANT-iron complexation or, more likely, through ANT interactions with iron-sequestering proteins such as ferritin and iron regulatory proteins [29]. Additional evidence to support the role of iron in ANT cardiotoxicity comes from the cardioprotective effects of the iron chelating agent dexrazoxane [19,29]. However, dexrazoxane may also act through the induction of hypoxia-inducible factor or by inhibiting the interaction of ANTs with *Top2β* [19,26,29].

More recent research has homed in on the interaction of ANTs, specifically DOX, with *Top2β* as a contributor to ANT CTX [19,26]. *Top2β* is one of two forms of topoisomerase 2 found in humans, the other being topoisomerase 2α (Top2α) (20). Top2α is primarily found in rapidly dividing cells and is bound by DOX, forming a DOX-Top2α-DNA complex which can induce DNA double-strand breaks (DSBs) [26]. Top2β, on the other hand, is predominantly found in cardiomyocytes, but is also bound by ANTs [26]. Zhang and colleagues, in a series of elegant experiments, found that *Top2*−/− mice were mostly resistant to the CTX of DOX, but *Top2*+/+ mice evinced increased expression of apoptosis promoter genes such as *Trp53inp1, Bax, Mdm2, and Fas*. Additionally, *Top2*−/− mice had 70% less ROS generation after exposure to DOX compared to Top2+/+ mice and had fewer mitochondrial structural changes. These results suggest that ROS generation in DOX CTX may be more related to DNA damage and transcriptomic changes rather than redox cycling.

The chemotherapeutic inhibitor of human epidermal growth factor receptor 2 (HER2 receptor) targets a proto-oncogene that is overexpressed in many breast cancers [30]. These therapies are monoclonal antibodies, such as trastuzumab and pertuzumab, as well as small molecule inhibitors, such as tucatinib and osimertinib (reviewed in greater detail in Swain). These therapies have drastically increased the survival rate of patients but come with an increased risk of both decreased left ventricular ejection fraction (LVEF) and congestive heart failure (CHF) [31]. The main reason for this increased risk is believed to be the inhibition of the cardioprotective signaling pathways of HER2 and neuregulin (NRG), its ligand [31]. Specifically, NRG-HER2 normally activates a pathway called PI3K/AKT, which increases the activation of nitric oxide synthase (NOS) and decreases levels of ROS [31]. The removal of this activation leads to increases in the levels of ROS and causes subsequent mitochondrial dysfunction and can lead to heart failure [31].

Other therapies that may increase ROS are VEGF inhibitors and VEGF receptor inhibitors, which inhibit angiogenesis by inhibiting the activation of the phospholipase C-protein kinase C-mitogen-activated protein kinase (PLC/PKC/MAPK) signal transduction cascade [32]. Some of the tyrosine kinase inhibitors (TKIs) that target the VEGFR for inhibition of angiogenesis in tumors also cause inhibition of the platelet-derived growth factor (PDGF)-activated PI3K/AKT pathway, which, as mentioned earlier, can increase the levels of ROS [31]. Additionally, sorafenib, a small molecule inhibitor of both PDGFR and VEGFR, was found to increase the levels of ROS in both the mitochondria and the cytosol along with decreasing the level of antioxidant enzymes [33].

The Bcr-Abl fusion protein is a result of a translocation between chromosomes 9 and 22, which creates a constitutively active tyrosine kinase, leading to chronic myelogenous leukemia [34]. The cardiotoxicity of Bcr-Abl TKIs is not negligible, with up to 8% of patients treated with ponatinib developing heart failure after treatment [35]. Potential mechanisms of this cardiotoxicity include the inhibition of the PI3K/AKT pathway, the inhibition of a multitude of other signaling pathways, and the potential induction of mitochondrial ROS production and dysfunction [35,36]. Additional adverse cardiac effects, including hypertension and vascular dysfunction, will be touched on later [32].

Some therapies that act more indirectly to increase the levels of ROS are antimetabolites, such as 5-fluorouracil (5-FU) and alkylating agents such as cyclophosphamide [37,38]. The most common side effect from 5-FU is coronary spasm, which is due to decreased vasodilatory molecules, as will be described later [37]. However, 5-FU also has direct effects on the endothelium, which may lead to the buildup of ROS, subsequent apoptosis, and endothelial and myocardial damage [37]. Cyclophosphamide, similarly, has been found to induce the production of ROS, decrease the concentration of antioxidant enzymes, and cause mitochondrial dysfunction [39]. It does this through its conversion in the liver to a toxic metabolite, acrolein, which can also induce the production of IL-6, a mediator of inflammation [40].

### 2.1. Inflammation and Immune Dysregulation

Inflammation and immune dysregulation play a key role in many cardiac pathologies, including heart failure, the primary phenotypic manifestation of CTX (HF) [19,41,42,43,44]. Inflammation and immune dysregulation often terminate in the elaboration of cytokines and chemokines, such as interleukin-6 (IL-6), which are sensed by the liver [28,45]. The liver subsequently produces C-reactive protein (CRP), which aids in upregulating the immune response, clearing extracellular nucleic acids, and activating complement [45]. An analysis by Burger et al. of the UCC-SMART cohort found that CRP was a risk marker of subsequent HF development in patients with previous cardiovascular disease (CVD) [46]. Additional trials have supported this connection by showing an increased prevalence of elevated CRP in both heart failure with reduced ejection fraction (HFrEF) and heart failure with preserved ejection fraction (HFpEF) [44].

There are various mechanisms that may lead to an increased production of cytokines, with some of the conserved pathways being activation of Toll-like receptors (TLRs), assembly of intracellular inflammasomes, and activation of the innate and adaptive immune system [19,42,47]. These cytokines can cause systolic and diastolic dysfunction, cardiomyocyte apoptosis, and interstitial fibrosis [44]. TLRs are a type of pattern-recognition receptor (PRR) that can generate inflammatory cytokines, interferon (IFN), and tumor necrosis factor-alpha (TNF-α) through activation of Myd88 [48]. An interesting connection is that oxidative stress, such as ROS production, can activate TLRs, suggesting a potential reciprocal relationship whereby ROS activate TLRs and TLRs can then cause more ROS to be produced [47,48]. Overexpression of TLRs is seen in both HF generally and in cardiotoxicity caused by ANTs, especially TLR2 and TLR4 [19].

Another pathway by which chemotherapeutics produce cytokines and cause immune dysregulation is activation of the NLRP3 inflammasome, which leads to the further activation of caspase-1, IL-1B and IL-18 formation, and the cleavage of gasdermin D (GSDMD), leading to apoptosis [49]. One of the chemotherapies that acts by this mechanism is immune checkpoint inhibitors, which typically inhibit programmed cell death ligand-1 (PD-1) or cytotoxic T lymphocyte antigen-4 (CTLA4) to increase the immune system’s responsiveness to tumor antigens [50]. This upregulation comes with the drawback of also increasing the expression of the NLRP3 inflammasome and IL-1B production with subsequent increases in macrophage activation and cardiac damage [42]. This may be one of the causes of the myocarditis that is sometimes noted acutely after administration of ICIs [50]. Another potential cause could be that there are some shared antigens between tumor cells and cardiomyocytes, leading to cross-reactivity and direct cytotoxic effects of the upregulated immune response [40,41,50].

CAR-T cell therapy is similar to ICIs in that there is greater activity of the immune system against tumor antigens, but the mechanism of cytokine induction is different [41]. The direct activity of CAR-modified T cells on their tumor antigen produces a robust T cell response and the subsequent release of high levels of cytokines, which is termed cytokine release syndrome (CRS) [41]. The primary mediator of the cardiotoxicity in CRS is thought to be IL-6, leading to cardiomyopathy and heart failure [41]. Other potential manifestations include myocardial infarction through a procoagulant mechanism and arrhythmias by disturbing electrolyte balance and inducing hypotension [51].

### 2.2. Endothelial and Vascular Injury

The endothelium plays a critical role in maintaining blood pressure, vascular permeability, and coagulability [52]. It does this by responding to and secreting a variety of substances in the bloodstream, including prostacyclin (PGI2) and nitric oxide (NO), which mediate vasodilation, and endothelin 1 (ET-1), which is a potent vasoconstrictor [52]. Prostacyclin can also inhibit platelet aggregation by antagonizing thromboxane A2, which is normally produced by platelets [29]. Dysfunction of these pathways can result in hypertension, increased vascular permeability, leukocyte adhesion and infiltration, and atherosclerosis [53]. In the context of chemotherapeutics, VEGF inhibitors, Bruton’s tyrosine kinase (BTK) inhibitors, antimetabolites, proteasome inhibitors, and radiotherapy are implicated in causing dysfunction and damage of the endothelium [31,37,54,55,56,57]. The most representative chemotherapy in this case is VEGF inhibitors [34].

VEGF inhibitors cause endothelial dysfunction by inhibiting multiple intracellular signaling pathways [31]. Typically, VEGF activates the PI3K/AKT pathway to increase the activity of endothelial nitric oxide synthase (eNOS), which produces NO [31,54]. VEGF can also activate the MAPK/cPLA2 and RAF/MEK/Erk pathways, producing PGI2 and factors involved in endothelial cell survival and homeostasis, respectively [31]. Inhibition of these pathways decreases the bioavailability of NO and prostacyclin and increases the concentrations of ET-1, leading to vasoconstriction and an increased risk of thrombosis [31,54]. Additionally, as mentioned previously, VEGF inhibitors can increase the concentration of ROS in endothelial cells and can disrupt calcium homeostasis, leading to endothelial damage [31].

Many of these pathways are also involved in the endothelial and vascular injury caused by the aforementioned chemotherapies as well. BTK inhibitors decrease the concentration of NO and inhibit PI3K [57]. Antimetabolites such as 5-fluorouracil decrease NO, directly cause cardiomyocyte damage, and increase ET-1, leading to coronary vasospasm [37]. Proteasome inhibitors lead to increased ROS formation and decreased NO, leading to dose-limiting hypertension [54]. Radiotherapy similarly increases ROS formation and decreases NO production, leading to endothelial apoptosis and fibrosis [33]. In most cases, the hypertension induced by these therapies is reversible on discontinuation [31].

### 2.3. Structural Remodeling and Fibrosis

Fibrosis has already been mentioned in several of the previously described mechanisms, and indeed fibrosis is often the culmination of several pathways including inflammation and endothelial dysfunction [23]. This can eventually lead to organ dysfunction and damage [23]. This fibrotic response is often driven by transforming growth factor-beta (TGF-β), which activates myofibroblasts and fibroblasts to secrete extracellular matrix [23]. A detailed discussion of all the pathways through which TGF-β acts is not possible in this review, due to their complexity and breadth (for more information, see the review by Massague).

Arrhythmias are a commonly seen consequence of cardiac fibrosis and remodeling, potentially because of the pro-arrhythmogenic conditions created by fibrosed tissue [23,57]. This is particularly relevant for BTK inhibitors such as ibrutinib, which are associated with an increased incidence of both atrial fibrillation (AF) and ventricular arrhythmias (VA) [31,57]. The fibrosed tissue is a result of the inhibition of PI3K and the induction of inflammatory cytokines by BTK inhibitors [57]. This tissue has an increased refractory period but a normal QT interval, predisposing to AF without increasing the risk of torsades de pointes [57].

Other pathways that can lead to fibrosis include the activation of macrophages by cytokines in inflammatory states, endothelial cell injury, sympathetic nervous system activation, and ROS production, which causes myocyte apoptosis and the release of DAMPs [19,23,56]. Radiation therapy is thought to trigger fibrosis mainly through the production of ROS, which leads to endothelial damage and the subsequent release of profibrotic molecules such as PDGF and TGF-β [56]. Thus, it is evident that many of these pathways are intertwined and many of the discussed chemotherapeutics act through multiple mechanisms, all contributing to their CTX.

### 2.4. Other Contributors to CVD

Emerging evidence indicates that there are other mechanisms by which chemotherapeutic and radiotherapies may impact the heart [8]. Irradiated tumor cells have been found to secrete a host of molecules with effects on cardiac cells [58]. Some of these molecules include previously mentioned DAMPs, which can activate TLR’s, cause inflammation, and subsequent fibrosis [58]. Other factors newly established as being part of the tumor secretome can have both cardioprotective and damaging effects, thus exposing both potential therapies as well as targets [58]. Another mechanism would be the inhibition of ion channels, specifically the delayed rectifier potassium channel, leading to QT interval prolongation as is seen in CDK4/6 inhibitors and certain TKIs such as sunitinib and sorafenib [31,59]. It must also be noted that certain patient-specific risk factors elevate their likelihood for developing cardiovascular disease as a result of chemotherapy treatment, such as a history of previous cardiovascular disease or certain genotypes that increase susceptibility [40].

Collectively, these mechanistic investigations have significantly improved our knowledge of cardiovascular damage caused by cancer therapy and have discovered several molecular pathways that could be targets for future treatments. However, it is challenging to ascertain the relative contribution of various pathways in clinical practice because a large portion of the current mechanistic information comes from preclinical models, translational research, or observational human data. The significant overlap among oxidative stress, inflammation, endothelial dysfunction, and fibrotic remodeling further implies that interrelated rather than distinct pathways are responsible for cardiotoxicity [60]. To find out how these biological insights might enhance risk classification and direct individualized surveillance and treatment plans, future prospective research combining mechanistic biomarkers with clinical phenotyping will be crucial.

Although these molecularly significant understandings clarify how cancer treatments cause cardiovascular damage, their practical utility is in guiding risk assessment and monitoring. Clinicians can identify high-risk patients, customize surveillance measures, and carry out prompt cardioprotective therapies by knowing how oxidative stress, inflammation, endothelial dysfunction, and fibrosis translate into diverse cardiovascular phenotypes. Therefore, the focus of the next section will change from the molecular causes of injury to their clinical manifestations and their use in modern cardio-oncology practice.

## 3. Clinical Phenotypes and Risk Stratification in Cardio-Oncology

### 3.1. Major Clinical Phenotypes of Cardiotoxicity

Manifestations of cancer therapy-related cardiovascular toxicity (CTR-CVT) are wide-ranging and encompass all layers of heart anatomy and aspects of heart function. Definitions for cardiotoxicity also vary across different governing bodies; Table 1 outlines the ACC definition for cardiotoxicity related to cancer treatment, which aligns well with the European Society of Cardiology [61]. Common presentations are well documented and similarities across various therapeutic treatments exist, which are summarized in Table 2.

#### 3.1.1. CTRCD

Cancer therapy-related cardiac dysfunction (CTRCD) is a common and concerning manifestation of cancer treatments. Associated with the first widely documented cardiotoxic chemotherapeutic, anthracycline, many therapeutics connected to cancers of bimodal age patient populations, including leukemia, lymphoma, breast cancers, and multiple myeloma, can lead to CTRCD [62]. The prevalence of CTRCD was found to be about 63 cases per 1000 patients in one year of follow-up. Most presentations occurred in the first 6 months of therapy cessation [64].

#### 3.1.2. Myocarditis

Myocarditis is a toxicity related to immune checkpoint inhibitors (ICIs) and cyclophosphamide, as well as other alkylating agents, with a 17% to 50% mortality rate in cancer patients. This high mortality rate is connected to the various complications from myocarditis, including fulminant disease, which can show hemodynamic instability, high-grade heart block, or significant arrhythmias [62,65]. ICI myocarditis is also linked with non-cardiac side effects of myositis and myasthenia gravis, so the presence of these two adverse effects should prompt cardiac evaluation [66].

#### 3.1.3. Vascular Toxicities

Vascular toxicities are a broad category of complications that include thrombosis, increased vasoactivity, and atherosclerosis related to treatment in cancer patients. This phenotypical cancer treatment effect can occur decades after remission, so long-term surveillance is critical, especially in younger patient populations [67,68].

Manifestations include: Vasospasms, Accelerated Atherosclerosis, Arterial Thrombosis, Vasculitis, Aneurysms, Dissections, and Hypertension.

Hypertension as a consequence of cancer treatment, especially in VEGF signaling pathway inhibitors and proteasome inhibitors. This is a common side effect in these therapies, occurring in between 11% and 45% of patients. It is important that therapy is started promptly, as untreated hypertension due to treatment can lead to cardiac dysfunction. Therapy typically can proceed with standard interventions unless systolic blood pressures spike to over 180 mmHg or diastolic blood pressures increase over 110 mmHg. In this case, the offending cancer treatment should cease until systolic pressures drop below 160 mmHg [61,68].

#### 3.1.4. Arrhythmias

The incidence of treatment-related arrhythmias is growing due to toxicities related to newer anti-cancer treatments as well as increasing patient-related risk factors like age at treatment and pre-existing inflammatory disease. The most prevalent arrhythmias are atrial fibrillation and QT prolongation, normally measured as a QTc of over 500 ms [40,69].

### 3.2. Patient-Related Risk Factors (Non-Modifiable vs. Modifiable)

Assessing patient risk factors is key in developing risk assessment tools for cardio-oncology and planning appropriate interventions. Many risk factors are shared between cardiovascular disease (CVD) and cancer, which leads to increasing overlap between these two previously disconnected pathologies. It is well-established that metabolic syndromes and obesity increase risk for both CVD and cancer, potentially through inflammation, oxidative stress, and changes in glucose management [68]. Additionally, hypertension has been linked with increases in cancer risk and mortality. An observational study that spanned 7 cohorts, three European countries, and over 575,000 patients indicated increased risk of cancer death in patients with hypertension as well as an increase in cancer incidence in men with hypertension [70]. As further studies continue to demonstrate a connection between cancer treatments and cardiac abnormalities, various governing bodies have released guidelines to support targeted care covered later in this paper. Table 3 highlights modifiable and non-modifiable patient risk factors related to cardiovascular toxicities [62].

### 3.3. Treatment-Related Risk Variables

Treatment-related risk factors in cardio-oncology are primarily related to total dosage, sequencing of various therapies, and direct drug toxicities.

#### 3.3.1. Cumulative Dosage

Cumulative dosage of anthracyclines is a well-established norm in assessing treatment-related risk of cardiac toxicities. The National Cancer Institute and the Children’s Oncology Group assess a dosage of between 0 and 250 mg/m^2^ Doxorubicin equivalent dosage as moderate risk and above 250 mg/m^2^ as high risk of heart failure. Chest radiation therapy should also be tracked independently and alongside anthracycline therapy. ASCO highlights moderate risk of heart failure as 15 to 29 grays; however, >15 grays with >100 mg/m^2^ of anthracycline is considered high risk. This type of additive effect is possible with other therapies as well, highlighting the need for further research into the long-term effects of combination therapy [69].

#### 3.3.2. Drug Sequencing

In addition to total dosage, improper drug sequencing is a risk factor for cardiac complications of treatment. Similar to cumulative dose, this is most relevant for anthracyclines. It is important to be careful when sequencing anthracyclines with anti-HER2 therapy, as anthracycline therapy followed by anti-HER2 therapy can increase risk for heart failure. This is also true in therapies combining different classes of ICIs and using multiple BRAF/MEK inhibitors in combination [69].

#### 3.3.3. Direct Drug Toxicities

The majority of drugs covered in this article are cardiotoxic on their own. The chief among these is anthracycline, which requires proper lifetime dosage tracking as cardiotoxicity is so great. It is also important to note when therapies cause red-herring symptoms. For instance, increases in peripheral edema caused by vascular damage from certain taxanes and anti-metabolites, or direct fluid retention from corticosteroids, can mimic fluid overload seen in heart failure [62].

### 3.4. Multimodal Risk Assessment Tools

Both targeted and generalized tools have been developed to assess risk for cardiotoxicity across different cancer modalities that consider both patient risk factors and treatment-related risk. These risk assessment tools include breast cancer, multiple myeloma, head and neck cancers, as well as immune checkpoint inhibitors, VEGF, and tyrosine kinase inhibitors, to name a few [71]. While these are helpful in specific scenarios, multiple broader risk assessment tools have been developed.

#### 3.4.1. ESC Guidelines on Cardio-Oncology

The most comprehensive risk assessment tool is the 2022 ESC Guidelines on Cardio-Oncology. This guideline was developed in collaboration with various organizations including the European Hematology Association, the European Society for Therapeutic Radiology and Oncology, and the International Cardio-Oncology Society. As an outcome, the guideline is an in-depth risk assessment from pre-treatment, covering six different classes of oncologic therapies, to appropriate interventions and contraindications of many known cancer therapies [62].

#### 3.4.2. Risk Stratification for This Guideline Focuses on the Following Factors

Risk stratification for this guideline focuses on the following factors:

- Previous CVD.
- Cardiac imaging.
- Cardiac biomarkers.
- Demographic and co-existing conditions known to be risk factors for CVD:
  ○ Age.
  ○ Hypertension.
  ○ Diabetes mellitus.
  ○ Hyperlipidemia.
  ○ Chronic kidney disease.
  ○ Proteinuria.
- Previous cardiotoxic cancer treatment.
- Lifestyle-related to CV risk factors:
  ○ Weight.
  ○ Smoking status.

Each criterion is scored for its likelihood of impact on the future development of CTR-CVT. For example, if the patient has hypertension and will receive anti-VEGF chemotherapy, it puts them at a high risk for cardiac-related complications. However, if the same patient was receiving a kinase inhibitor for CML, their hypertension would carry a medium risk level of future CTR-CVT complications. The overall risk level for the patient is then defined by the highest risk score they received in pre-treatment or if they have multiple risk factors that can increase their assigned risk level. This risk level can be used to guide proactive monitoring schedules and, in high-risk scenarios, alternative treatment options. Diagram 1 [25,50,63] outlines the risk assessment flow based on these guidelines.

Although the guideline covers additional therapies, the pre-treatment risk assessments for various at-risk therapies, including ICI therapy, alkylating agents, and taxanes, are not present due to limited data. Similar structures can still apply in history and diagnostic testing.

**Figure 1:**
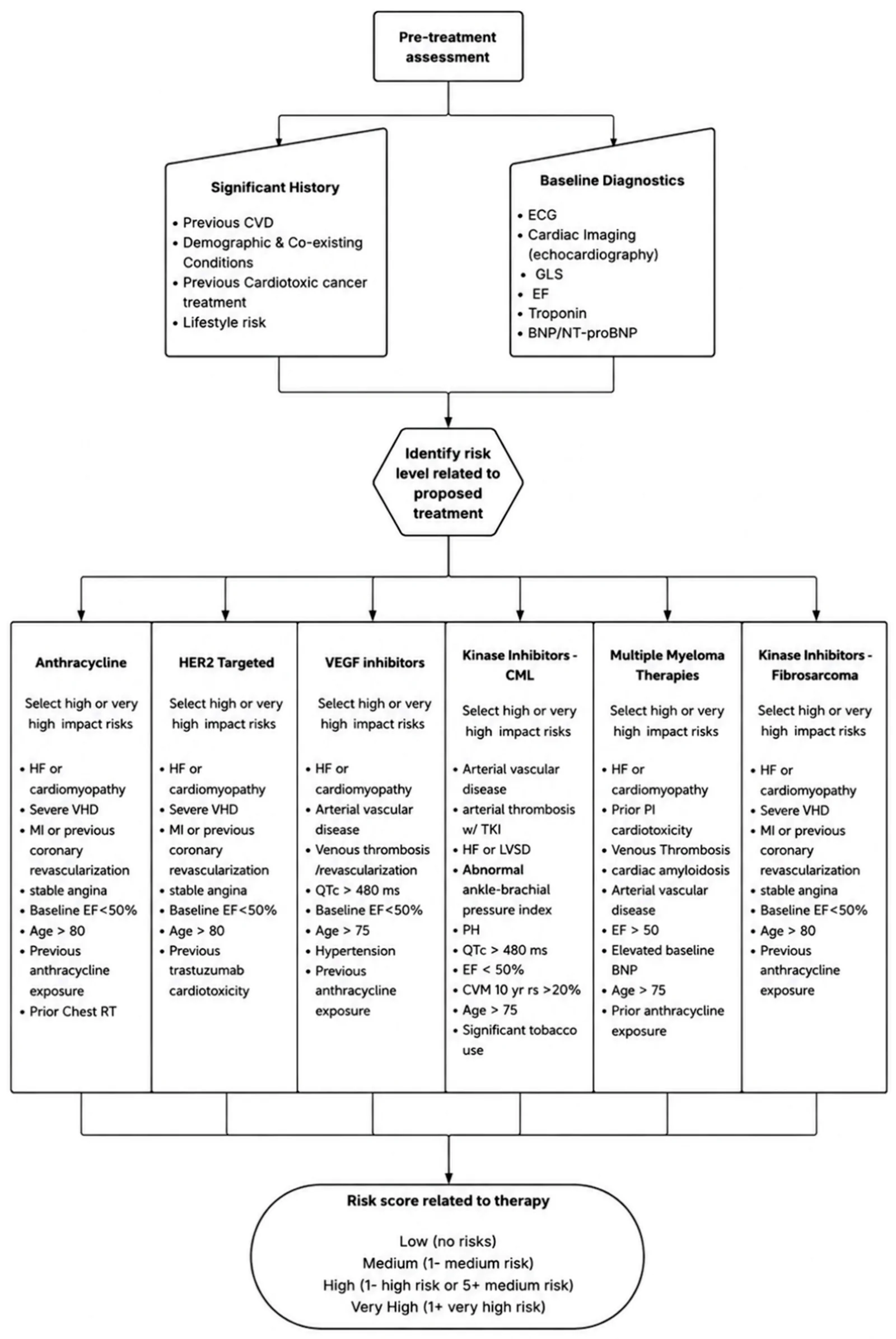
Proposed risk stratification assessment for pre-treatment with anthracyclines, HER2 therapies, VEGF inhibitors, kinase inhibitors (CML and Fibrosarcoma), and multiple myeloma therapies. This diagram was developed from 2022 ESC guidelines on cardio-toxicity.

Cardiovascular risk stratification has made significant strides, yet there are still significant obstacles. With reported C-statistics typically ranging from 0.60 to 0.70, the majority of currently available prediction models exhibit only minor discriminative performance, which limits their capacity to correctly identify individuals who may eventually develop cardiotoxicity. Concerns about the generalizability and practical usefulness of several models have also been raised by their inadequate external validation across a variety of cancer populations, treatment regimens, and healthcare settings [16,17]. Additionally, the molecular mechanisms that contribute to an individual’s susceptibility to cardiovascular injury are only partially incorporated into current frameworks, which mostly focus on clinical features and treatment-related variables. Emerging methods that combine artificial intelligence, multi-omics profiling, and molecular biomarkers have demonstrated promise for enhancing personalized risk prediction; however, these technologies are still in the research stage and their added benefit over current models has not yet been determined. Before these methods may be regularly included in cardiovascular risk assessment and surveillance, more prospective validation, consistent implementation, and proof of clinical value are needed (Figure 1).

The main guidelines for cardiovascular risk assessment and surveillance in cardio-oncology from the 2022 ESC, ASCO, ICOS, and ACC/AHA guidance publications are contrasted in Table 4. The scope, surveillance recommendations, biomarker use, and follow-up tactics of these organizations differ significantly, despite the fact that they share many fundamental ideas. These discrepancies are outlined to make practical application simpler.

## 4. Surveillance Strategies Across the Cancer Care Continuum

### 4.1. Evolution of Cardiovascular Surveillance

With some remarkable advancements in cancer treatment over the last three decades, cancer-related mortality has declined, which is reflected by increased numbers of cancer survivors [72]. As a result, cardio-oncology has gained significant importance because of its impact not only on the type of cancer therapy but also on the long-term outcomes of cancer survivors.

Cardiovascular surveillance in cardio-oncology has evolved from traditional, phenotype-based monitoring such as left ventricular ejection fraction (LVEF) to a more integrated, risk-adapted, and mechanism-informed approach. The current approach is multimodal and emphasizes early detection of myocardial injury using clinical assessment and other tools such as advanced imaging and circulating biomarkers.

Current surveillance strategies vary across professional societies, with the European Society of Cardiology 2022 guidelines providing a comprehensive, risk-adapted framework, which remains the only unified cardio-oncology guideline [62], whereas other available recommendations are more fragmented and disease-specific.

Table 4 lists the significant main parallels and discrepancies between the 2022 ESC, ASCO, ICOS, and ACC/AHA recommendations for cardiovascular risk assessment and surveillance in order to put these recommendations in a more comprehensive clinical context. While pointing out disparities in scope, surveillance suggestions, and data supporting present practice, this comparison also identifies areas of agreement.

### 4.2. Baseline Cardiovascular Assessment

#### 4.2.1. Pre-Treatment Risk Categorization

Baseline cardiovascular assessment in cardio-oncology has also evolved from pre-treatment clearance to a structured risk stratification strategy that involves baseline cardiovascular conditions, surveillance intensity and preventive strategy across the treatment continuum.

The most notable shift in modern cardio-oncology is moving from listing baseline risk factors, i.e., age, sex, history of CVD, previous cardiotoxic therapies or radiation exposure, and lifestyle, to the HFA-ICOS risk assessment tool, which integrates patient-related factors (age, cardiovascular disease, metabolic comorbidities), prior exposure, and therapy-specific cardiotoxicity into a unified scoring system [68]. This allows us to stratify cancer patients into low, medium, and high risk of cardiovascular complications before treatment and to have a more personalized approach. However, it is worth noting that the likelihood of occurrence of a complication and its severity are two different entities, and the timeline may also be different [62].

#### 4.2.2. Baseline Echocardiography with Strain

Baseline cardiac imaging has similarly evolved from documenting normal LVEF to a comprehensive functional assessment of myocardial performance. Assessment of LVEF and GLS should be performed in all patients undergoing TTE prior to initiation of cardiotoxic cancer therapy to aid in cancer therapy-related cardiotoxicity risk stratification and to detect significant changes during treatment (Class I recommendation from the European Society of Cardiology cardio-oncology guidelines) [62].

Although left ventricular ejection fraction (LVEF) is still a standard measure, its ability to detect early heart damage is limited. A noticeable decrease in LVEF is only seen after significant heart injury has already occurred. In comparison, myocardial deformation imaging, particularly global longitudinal strain, enables detection of subclinical dysfunction at an earlier stage [73,74]. In a systematic review and meta-analysis, Kashif Kalam et al. showed that global longitudinal strain (GLS) is a stronger predictor of mortality and major adverse cardiac events than left ventricular ejection fraction (LVEF) [75]. This signifies baseline imaging as a tool for early risk identification rather than a measure of established dysfunction.

#### 4.2.3. Baseline Biomarkers

The role of cardiac biomarkers remains crucial in cardio-oncology and has transitioned from mere detection of myocardial injury to prospective risk stratification and longitudinal tracking. Cardiac biomarkers, such as troponin (cTn I/T) and natriuretic peptides (BNP or NT-proBNP), remain the most extensively studied and validated biomarkers and assist in baseline cardiovascular risk stratification in patients undergoing potentially cardiotoxic cancer therapies, such as anthracyclines, HER2-targeted therapies, VEGF inhibitors, proteasome inhibitors, immune checkpoint inhibitors, and cellular therapies (CAR-T and TIL) [76]. Elevated baseline high-sensitivity troponin and pro-BNP are indicators of who will develop cardiac dysfunction and also predict the risk of lack of recovery after guideline-based heart failure therapy [77]. In a prospective cohort study, Noemi Pavo et al. showed that elevated NT-proBNP and hs-troponin levels were strong independent predictors of all-cause mortality in patients with cancer [78]. Due to the relatively inexpensive nature and ready availability of such testing, it is a Class I recommendation for cardiac biomarker assessment prior to potentially cardiotoxic therapies in the European Society of Cardiology cardio-oncology guidelines [62].

In a recent systematic review (2023), novel biomarkers such as microRNAs and genetic variants (e.g., SNPs) have demonstrated potential in predicting cardiotoxicity, but their clinical role remains uncertain, with further studies required to validate their prognostic significance [79].

Significant restrictions still exist despite their well-established use in baseline cardiovascular evaluation. Elevations in troponin and natriuretic peptides may indicate underlying cardiovascular disease, renal failure, or other comorbidities and are not specific to cardiac harm caused by cancer therapy. Furthermore, whereas current guidelines encourage baseline biomarker evaluation, there is still no evidence that biomarker-guided surveillance alone improves long-term cardiovascular outcomes. Similar to this, new biomarkers, such as microRNAs and genetic variations, have shown promising initial results but do not yet have standardized assays, external validation, or prospective studies proving their therapeutic value [79]. As a result, a lot of biomarker-guided surveillance recommendations are still based more on expert opinion than on solid randomized research.

#### 4.2.4. Assignment to Surveillance Tier

The integration of clinical risk factors, imaging findings and biomarkers leads to assignment to a surveillance tier, which represents ESC and HFA frameworks that stratify patients into risk categories (low, moderate, high) that directly determine the frequency of imaging, need for biomarker monitoring, intensity of follow-up, and consideration of cardioprotective therapy. Therefore, the guidelines recommend that low-risk patients may proceed with routine oncologic care and standard surveillance, whereas those at moderate, high, or very high risk warrant early cardiology or cardio-oncology evaluation before initiation of therapy to improve cardiovascular outcomes [62].

This represents a significant change from currently established fixed interval monitoring strategies to a risk stratification pathway-driven model where baseline assessment directly defines who needs specialist engagement from the start as well as how patients are tracked.

Additionally, baseline assessment increasingly identifies patients who may benefit from early preventive interventions, including renin–angiotensin system inhibitors, beta-blockers, or dexrazoxane in selected high-risk populations, although evidence remains heterogeneous [80,81].

### 4.3. Monitoring During Active Therapy

#### 4.3.1. Surveillance Intervals Based on Risk

Surveillance during active cancer therapy should be tailored to both baseline cardiovascular risk and the cardiotoxic characteristics of the anticancer medication. Emerging data shows the importance of strategy-sensitive surveillance. In the SUCCOUR trial, GLS-guided monitoring improved earlier detection of subclinical dysfunction and earlier initiation of cardioprotective treatment compared with LVEF-guided approaches. Meta-analytic studies also show that early reductions in GLS predict subsequent cardiotoxicity, which highlights the need for closer interval monitoring in higher-risk patients [82].

This risk-adapted surveillance approach is summarized in Section 4.4.4, which shows how surveillance intensity varies based on therapy, cardiovascular risk, biomarker findings, and clinical status during the pre-treatment, active treatment, end-of-therapy, and long-term survivorship phases.

The 2022 ESC cardio-oncology guideline has altered practice away from uniform monitoring toward risk-adapted, therapy-specific surveillance, in which the frequency and modality of assessment reflect anticipated toxicity burden.

In anthracycline-treated patients, surveillance is based on baseline risk and cumulative dose. The guidelines recommend baseline assessment followed by repeat evaluation during therapy, with higher-risk patients getting imaging and biomarker assessment approximately every 2–3 cycles or every 3 months, particularly at higher cumulative doses (Class 1), whereas lower-risk patients may be monitored less frequently. Importantly, surveillance is not solely time-based but may be escalated earlier if abnormalities in GLS or cardiac biomarkers are detected, reflecting a dynamic monitoring strategy [62].

For HER2-targeted treatments, it is advised to evaluate left ventricular function at baseline and every three months throughout treatment (Class 1). For patients with a higher baseline risk or early signs of dysfunction, monitoring may be done more often, such as every 6 to 8 weeks. Although specific timing is individualized rather than fixed in guidelines [62].

In patients receiving VEGF inhibitors, surveillance is more explicitly risk-stratified. The guideline suggests that in moderate-risk patients, echocardiography may be performed every 4 months during the first year (Class 2b), whereas in high- and very high-risk patients, imaging is recommended every 3 months (Class 2a), with natriuretic peptide monitoring at baseline, at 4 weeks after therapy initiation, and, subsequently, every 3 months (Class 2a) [62].

For immune checkpoint inhibitors, surveillance is not routine interval-based but rather symptom- and risk-driven, given the potential for early and rapidly progressive myocarditis [83]. Hence, guidelines propose baseline assessment and a low threshold for early evaluation with troponin, ECG, and imaging during the initial cycles of therapy, particularly in higher-risk patients [62].

In contrast to passive periodic testing, current recommendations emphasize that monitoring should operate as an active therapeutic process connecting early identification to intervention. However, because few randomized studies have directly evaluated various monitoring systems, the ideal frequency and level of surveillance are still unknown. As a result, many surveillance schedules suggested in current guidelines are based more on expert consensus and observational data than on high-quality comparative evidence, underscoring the necessity of prospective trials to identify the most economical and successful surveillance strategies [62,84]. Guideline recommendations should be integrated with individualized clinical judgment in surveillance decisions until stronger prospective evidence is available. Treatment-specific cardiovascular risk, baseline cardiovascular status, imaging and biomarker findings, and changing clinical symptoms should all be considered.

#### 4.3.2. GLS-Guided Early Detection

Global longitudinal strain (GLS) enables detection of myocardial injury during therapy prior to a reduction in a left ventricular ejection fraction (LVEF), thereby transitioning monitoring from static observation to early, response-guided detection. Observational studies and meta-analyses show that relative reductions in GLS during treatment precede and predict subsequent cardiotoxicity, with a threshold of approximately 13–15% reduction linked to later decline in LVEF, establishing the foundation for current definitions of subclinical dysfunction [74].

Randomized research further substantiates the clinical significance of this methodology. In the SUCCOUR trial, GLS-guided surveillance led to earlier identification of myocardial dysfunction and prompt initiation of cardioprotective therapy, in contrast to standard LVEF-guided monitoring. This demonstrates that the surveillance modality can influence the timing of intervention [82]. Long-term follow-up revealed improved recovery of left ventricular function at three years; however, differences in key outcomes remained minimal [85].

Overall, GLS plays a significant role in modern multimodal surveillance by facilitating the early identification of possibly treatable myocardial dysfunction. However, there are still significant unanswered issues about whether earlier therapy consistently results in better long-term cardiovascular outcomes, the ideal threshold for intervention, and inter-vendor variability in strain assessments. Further multicenter randomized studies are required to properly clarify GLS’s function in ordinary clinical practice, even though the evidence now available supports its incorporation into surveillance systems.

#### 4.3.3. Biomarker-Triggered Escalation Protocols

Biomarker-triggered escalation protocols represent an important evolution in surveillance during active therapy, shifting biomarkers from passive indicators of injury to tools that can prompt earlier imaging, closer follow-up, and selective cardioprotective intervention.

In the ICOS-ONE trial, treating patients with enalapril only after troponin increased worked in practice, but outcomes were similar to treating everyone, so this approach may not be clearly better, suggesting that biomarker-guided escalation is clinically practical but not yet clearly superior as a stand-alone strategy [86]. More recently, Cardiac CARE showed that high-sensitivity troponin I-guided candesartan and carvedilol did not prevent decline in ventricular function, underscoring that biomarker rise alone may be insufficient to define an effective intervention pathway [80].

However, in a recent randomized trial, NT-proBNP-guided therapy was feasible and safe, and led to earlier initiation of cardioprotective treatment, with a modest short-term improvement in LVEF, supporting the concept that biomarker-guided escalation may help identify patients who benefit from early intervention [87].

Together, these studies suggest that biomarker-triggered escalation is best understood as part of a broader multimodal surveillance strategy, while optimal thresholds, timing, and treatment responses remain active areas of investigation.

#### 4.3.4. High-Risk Therapy Monitoring Pathways

As discussed in the preceding sections and summarized in Table 1, the level of surveillance during active therapy is determined by the patient’s baseline risk category. High-risk patients need more intensive and flexible monitoring pathways. In this cohort, imaging and biomarkers are incorporated earlier and more regularly, with a reduced threshold for escalation predicated on variations in strain, biomarker elevation, or clinical condition.

Surveillance is closely related to making clinical decisions in real time. For example, if there are early signs of myocardial injury, cardioprotective therapy, more involvement from cardiology, or changes to cancer treatment may be needed (Table 5).

### 4.4. Post-Therapy and Survivorship Surveillance

#### 4.4.1. Late Cardiomyopathy Detection

Chemotherapy-related cardiovascular toxicity does not end with treatment; late-onset cardiomyopathies represent a delayed manifestation of chemotherapy-related cardiac injury. This is most evident in dose-dependent myocyte injury and impaired repair mechanisms following anthracycline exposure years after treatment.

This long latency has been consistently demonstrated in large survivor cohorts. In the Childhood Cancer Survivor Study [88], adult survivors exposed to cardiotoxic therapy had significantly increased risks of heart failure, myocardial infarction, and other cardiovascular events compared with controls, establishing that cardiotoxicity persists decades beyond treatment. A subsequent cross-sectional analysis within the same cohort further demonstrated a high prevalence of asymptomatic cardiac disease among survivors, highlighting that a substantial proportion of patients carry subclinical disease long before clinical presentation [3].

In another community-based cohort study looking beyond the childhood cancer population, Saro H. Armenian et al. [89] demonstrated increased long-term cardiovascular disease in survivors of adult-onset cancers, expanding the concept of delayed cardiotoxicity to larger cancer populations. Previous survivorship analyses have also shown that long-term survivors experience a high prevalence of chronic cardiovascular morbidity, supporting the idea that cardiotoxic damage develops gradually over time rather than resolving after therapy is finished [55].

**Figure 2:**
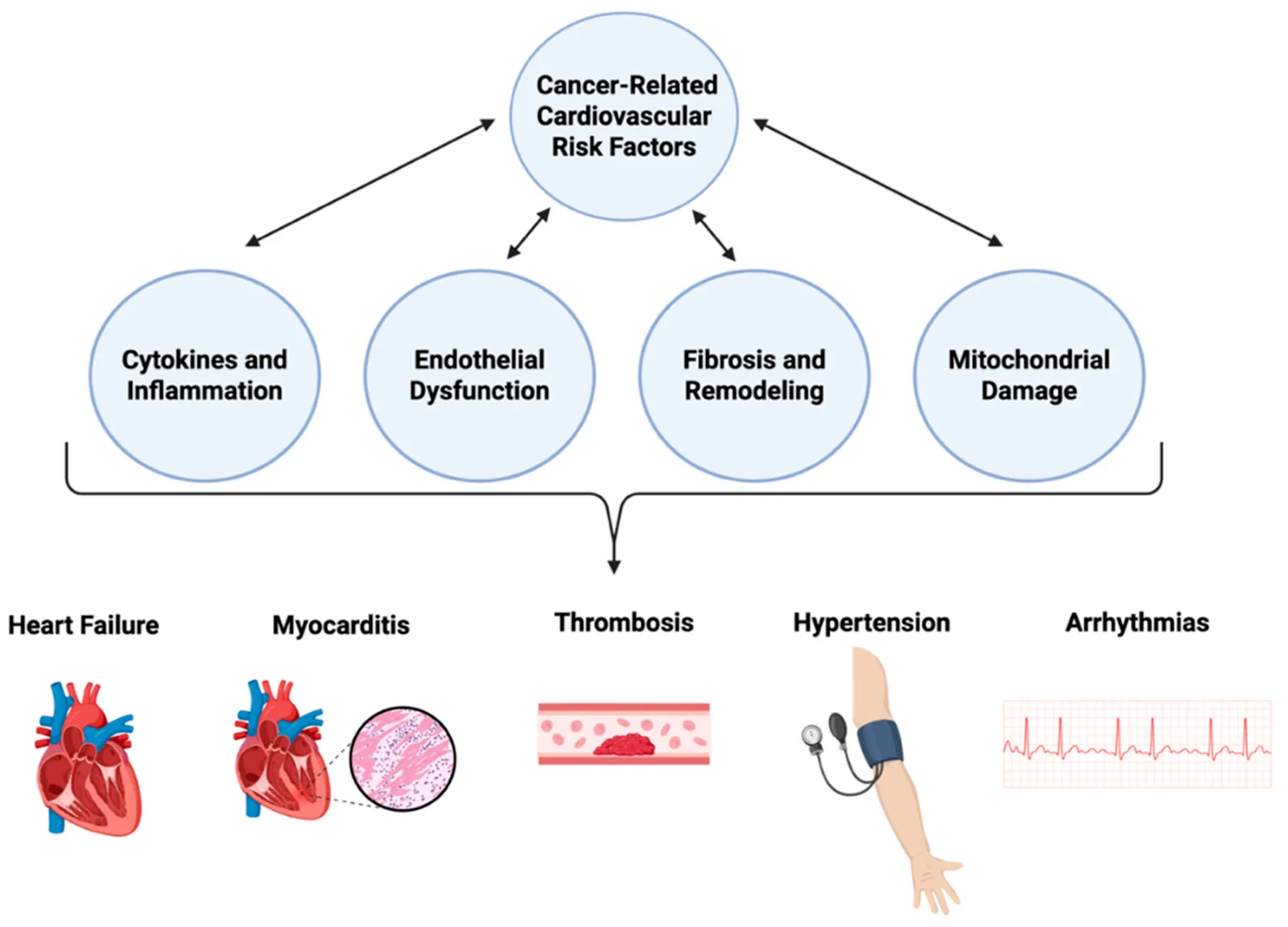
Mechanistic pathways of cancer therapy-induced cardiovascular injury. Created in BioRender. Beech, W. (2026) https://BioRender.com/89hwxfq, accessed on 2 August 2026.

Together, these investigations define late cardiomyopathy as a silent, progressive disease that requires lifetime, risk-adapted monitoring. According to the 2022 ESC Cardio-Oncology Guidelines, at-risk survivors should undergo recurrent imaging and early diagnosis of asymptomatic impairment to enable timely initiation of cardioprotective intervention. This approach to cardiovascular surveillance is summarized in Figure 2.

#### 4.4.2. Radiation-Related Delayed Vascular Disease

Radiation-related cardiovascular disease represents a distinct late toxicity characterized by gradual injury, causing macrovascular and microvascular dysfunction due to endothelial injury, inflammation, DNA damage, and oxidative stress [90,91]. Unlike anthracycline cardiomyopathy, which primarily affects myocardial function, radiation-induced injury manifests predominantly as vascular disease with delayed clinical expression.

Clinical studies corroborate these findings and emphasize the temporal progression of vascular damage. Preliminary observational studies, including patients receiving head and neck radiotherapy, indicated a markedly increased prevalence of carotid artery disease, often manifesting years after treatment [92]. Subsequent imaging studies have expanded these findings by detecting subclinical vascular alterations at an earlier stage, such as increased carotid intima-media thickness and early-onset atherosclerosis in chronically exposed individuals, suggesting that vascular injury initiates long before apparent clinical manifestations and evolves over time [93].

Long-term survivorship data demonstrate that vascular toxicity is not limited to radiation exposure but also occurs after chemotherapy, resulting in late vascular complications in cancer survivors. In testicular cancer survivors observed for more than twenty years following cisplatin-based chemotherapy, vascular assessments indicated heightened arterial stiffness and features suggestive of accelerated vascular aging, implying that treatment-related vascular damage is both progressive and persistent [94]. These results highlight the significant vascular implications of cancer therapy outside the heart and confirm that vascular damage persists and advances far after the conclusion of treatment.

Overall, these results underline the necessity of long-term, risk-adapted surveillance and aggressive control of modifiable cardiovascular risk variables by supporting the idea that early subclinical vascular injury gradually develops into clinically relevant illness. Nevertheless, it is challenging to identify the best surveillance tactics because a large portion of the evidence that is currently available comes from observational cohorts with varied treatment exposures and extended follow-up periods. Further prospective studies are required to determine which survivors benefit most from vascular imaging and to create evidence-based screening intervals.

#### 4.4.3. Long-Term Survivorship Clinics

The fact that cardiovascular toxicity evolves over the year after cancer therapy has led to the development of structured survivorship care models, particularly cardio-oncology clinics, to deliver longitudinal follow-up. This need arises from consistent evidence revealing substantial burden of late cardiovascular disease among cancer survivors, including both major cardiovascular events and subclinical dysfunction that remain undetected in routine care.

Recent multidisciplinary models have begun to enhance the provision of survivorship care within the cancer care continuum, emphasizing a patient-centered approach and a prevention-oriented policy. A comprehensive cardio-oncology roadmap includes integrated care models featuring risk stratification, multimodal surveillance (including imaging and biomarkers), and coordinated management across oncology, cardiology, and primary care, emphasizing continuous follow-up rather than isolated visits [95].

A comprehensive statewide study of Dutch healthcare experts, including cardiologists, radiation oncologists, medical oncologists, and hematologists, revealed that while awareness of cardio-oncology is on the rise, practical implementation remains insufficient. A significant number of responders were unaware of current guidelines, perceived a deficiency in their understanding to handle cardio-oncology patients, and emphasized the necessity for more training, practical direction, and improved interdisciplinary collaboration [96].

Dedicated multidisciplinary cardio-oncology programs at the institutional level have shown that structured survivorship clinics can effectively integrate risk-stratified surveillance and preventive care into routine oncology follow-up. Reports have shown that these clinics can detect subclinical cardiotoxicity earlier, improve care coordination, and have high patient-reported value, especially in high-risk groups like childhood cancer survivors [97].

Collectively, these results point to a shift toward multidisciplinary, long-term survivorship models that incorporate cardiovascular treatment at every stage of the cancer process. However, the majority of the information now available comes from observational research, expert agreement, and institutional experiences; there is little prospective data showing improvements in long-term cardiovascular outcomes. Access to specialized cardio-oncology services, resource availability, and variability in care pathways are just a few of the significant obstacles that still exist. Standardized survivorship models should be assessed in a variety of healthcare settings in future research to ascertain their efficacy, scalability, and affordability.

#### 4.4.4. Transition to Primary Cardiovascular Care

The transition from specialized cardio-oncology follow-up to primary cardiovascular care represents a critical but often underdeveloped phase of survivorship. Cancer survivors carry a 2–5-fold higher risk of cardiovascular mortality compared with the general population, underscoring the need for continued long-term cardiovascular surveillance beyond oncology-directed care [68]. Moreover, expert recommendations emphasize that all survivors should undergo at least annual cardiovascular risk assessment and optimization of risk factors, with additional imaging and surveillance in higher-risk individuals [68].

A major barrier to effective transition is the lack of standardized care models and clinician preparedness [96]. Modern frameworks emphasize the importance of a shared-care model, wherein primary care physicians and general cardiologists collaboratively manage long-term care. This approach is further strengthened by thorough documentation of prior medical care, tailored risk stratification, and the availability of cardio-oncology expertise as required [95].

The best way to go from specialized cardio-oncology services to primary cardiovascular care is still poorly understood, despite the rising understanding of the significance of shared-care models. The recommendations that are already in place are largely based on expert consensus rather than comparative effectiveness research, and there is significant variation in how these recommendations are implemented across healthcare systems. Future research should continue to prioritize creating standardized transition pathways and assessing their effects on long-term patient outcomes.

**Figure 3:**
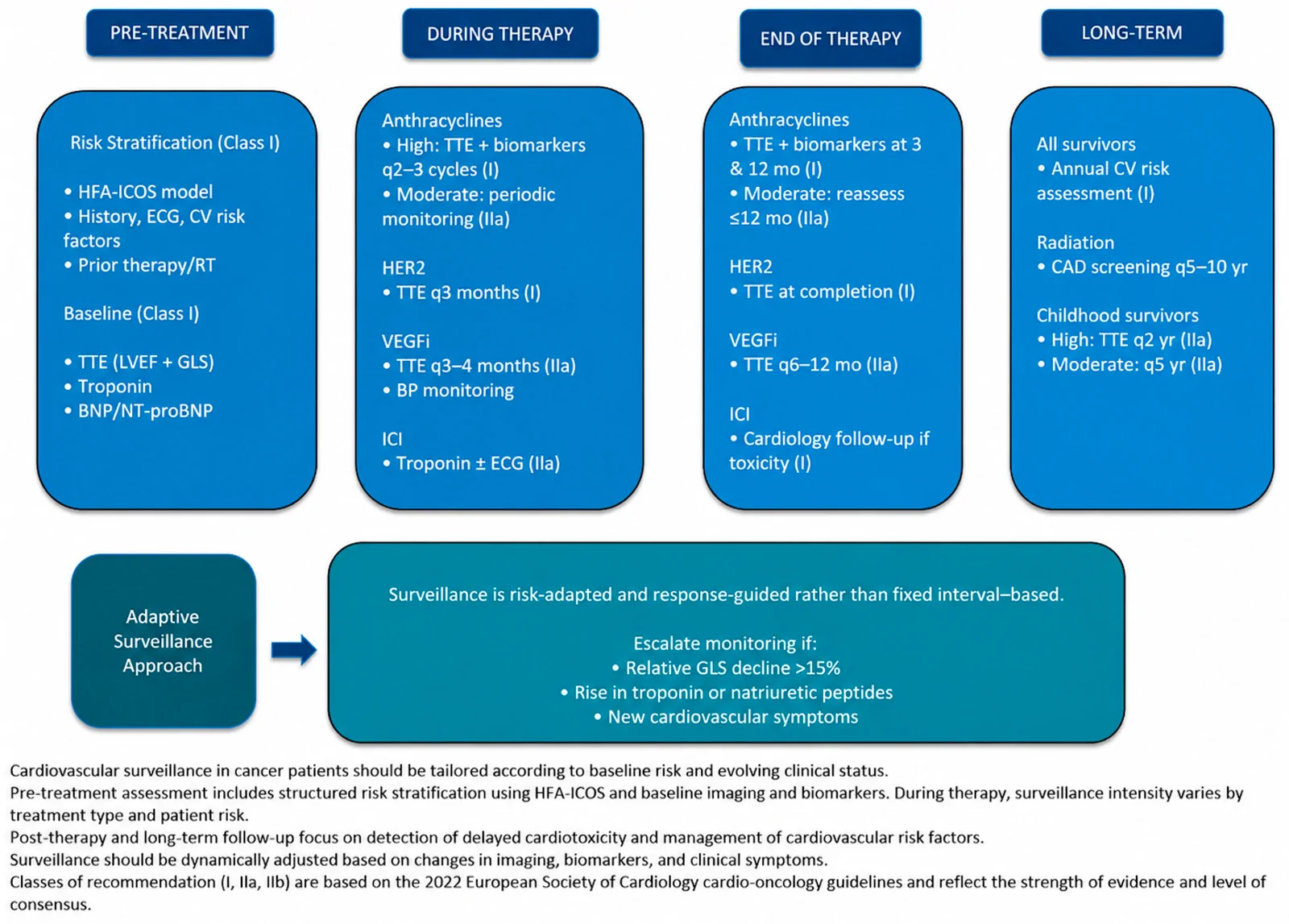
Timeline of Cardiovascular Surveillance in Cancer Patients. Created in BioRender. Beech, W. (2026) https://BioRender.com/94fqerj, accessed on 2 August 2026.

Cardiovascular surveillance should be tailored based on treatment phase, baseline cardiovascular risk, therapy-specific toxicity, biomarkers, imaging findings, and changing clinical status in order to give clinicians a useful framework (Figure 3). 

## 5. Future Directions and Precision Cardio-Oncology

The future of cardio-oncology lies in transitioning from static surveillance toward mechanistic-based surveillance that enables earlier detection, improved individualized risk prediction, and earlier intervention. While the field has grown recently due to developments in molecular profiling, dynamic biomarker surveillance, multimodal imaging, and artificial intelligence, many of these technologies are still in the research stage and need prospective validation before being widely used in clinical settings. Recently published data highlight advances in molecular profiling, dynamic biomarker surveillance, multimodal imaging, and artificial intelligence that may reshape cardio-oncology care.

### 5.1. Mechanism-Guided Risk Models

Current risk models rely on static clinical variables and treatment exposures, which do not capture the dynamic and biologically heterogeneous nature of cardiotoxicity. Future approaches may incorporate mechanistic domains—including inflammation, oxidative stress, endothelial dysfunction, and fibrotic remodeling—into integrated risk prediction models. Recent work emphasizes the importance of dynamic risk stratification using longitudinal biomarker trajectories and imaging parameters such as global longitudinal strain (GLS) [68,81]. In addition, targeting dynamic biomarkers is an emerging area with significant potential. Mechanism-guided models may allow earlier identification of subclinical injury, refinement of surveillance intervals, and better differentiation of cardiotoxic phenotypes, ultimately supporting more tailored therapeutic strategies. Before normal clinical use, prospective validation in a variety of patient populations is still required, and their incremental utility beyond current clinical risk models is still unknown.

### 5.2. Multi-Omics and Molecular Biomarkers

Multi-omics approaches have the ability to redefine cardiovascular risk assessment at the molecular level. Genomic studies have identified susceptibility loci associated with anthracycline-induced cardiomyopathy and variability in drug metabolism [18]. Proteomic and metabolomic profiling further allow detection of early changes in inflammatory, oxidative, and metabolic pathways before overt cardiac dysfunction develops. Circulating biomarkers reflecting inflammation–fibrosis signaling and mitochondrial injury may provide greater sensitivity than traditional markers such as troponin and natriuretic peptides [98]. These methods are still mostly experimental, despite early research showing encouraging outcomes. Their adoption into ordinary clinical practice is now hampered by issues such as poor external validation, a lack of standardized assays, unclear cost-effectiveness, and insufficient prospective data.

### 5.3. Artificial Intelligence and Advanced Imaging

In cardio-oncology, artificial intelligence is an emerging tool that can help identify high-risk patients earlier and support more personalized care. Global longitudinal strain–based echocardiography has been shown to reduce progression to abnormal left ventricular ejection fraction in patients receiving chemotherapy [81,99]. Cardiac CT and cardiac MRI also allow earlier and more precise detection of vascular and myocardial injury, improving identification of subclinical cardiomyocyte damage and supporting more accurate risk stratification [100,101,102,103,104]. On the other hand, prediction models based on artificial intelligence are still a developing field of study. By combining imaging, biomarkers, and clinical factors, machine learning algorithms have shown promising prediction performance; nevertheless, most models have not yet shown improved clinical outcomes in prospective studies and have received little external validation. As a result, rather than taking the place of well-established surveillance techniques, AI should now be seen as an additional research instrument. Machine learning-based prediction models can integrate biomarkers, clinical variables, and imaging data to improve both primary and secondary prevention of cardiovascular complications related to cancer therapy.

### 5.4. Preventive and Cardioprotective Strategies

The trend in modern medicine has shifted toward primary prevention, and in cardio-oncology, this area is still evolving. This has led to the development of tools that help identify patients at higher cardiovascular risk earlier in their treatment course. Cardioprotective strategies include early initiation of ACE inhibitors and beta-blockers, which have been shown to limit declines in left ventricular systolic function in high-risk populations. Ongoing studies are evaluating earlier use of cardioprotective therapies as well as newer agents targeting inflammation, oxidative stress, and fibrosis. One specific agent, dexrazoxane, reduces the incidence and severity of anthracycline-induced cardiotoxicity in patients at increased risk for cardiac dysfunction without compromising antitumor efficacy and remains the standard of care for preventing tissue injury from Doxorubicin extravasation. As of right now, dexrazoxane is a significant FDA-approved cardioprotective medication that may reduce anthracycline-associated cardiotoxicity in carefully chosen patients, and it is still a common part of treatment in these situations [105,106,107]. Research is currently being conducted to see whether cardioprotective techniques can be applied to larger patient groups and whether treatments that target oxidative stress, fibrosis, and inflammation may offer further advantages.

## 6. Conclusions

Cardio-oncology has become a vital field due to advancements in cancer treatment, with an increasing number of people at risk for long-term cardiovascular events. Cancer therapy-related cardiovascular toxicity consists of a spectrum of various disorders including myocardial, inflammatory, electrophysiologic, and fibrotic disorders. These disorders are caused by interrelated mechanisms such as immune dysregulation, endothelial injury, and maladaptive structural remodeling. These mechanistic insights emphasize the shortcomings of traditional exposure-based surveillance while supporting continued attempts to develop biologically informed and mechanism-guided approaches to cardiovascular risk assessment. Clinical risk assessment, circulating biomarkers, and new imaging modalities, such as global longitudinal strain, are all part of modern monitoring systems that are increasingly utilized to improve the early diagnosis of subclinical cardiovascular injury. Optimizing monitoring intervals, improving risk classification, and identifying individuals most likely to benefit from preventive and cardioprotective medications are some of the significant difficulties that still need to be addressed. Multi-omics profiling, molecular biomarkers, AI-powered prediction models, and sophisticated imaging methods are examples of emerging technologies that have shown encouraging potential to further customize cardiovascular risk assessment. However, before being routinely used in clinical settings, many of these strategies are still in the research stage and need more prospective validation. To improve survivorship care, lower long-term cardiovascular morbidity, and maintain oncologic efficacy, cardiology, oncology, imaging specialists, and translational researchers must continue to collaborate across disciplines.

## Figures and Tables

**Table 1:** ACC consensus statement for cancer therapy-related cardiovascular toxicities.

Phenotype	Criteria
Symptomatic CTRCD	Signs of heart failure Fluid overload Inadequate perfusion
Asymptomatic CTRCD	Mild EF ≥ 50% Reduction in baseline GLS >15% Or Rise in troponins/natriuretic peptides Moderate EF between 40 and 49% Severe EF < 40%
Myocarditis	Combination of testing and clinical findings congruent with myocarditis including: Major Criteria Definite cardiac MRI findings Minor Criteria Clinical syndrome Arrhythmias Decrease in EF Regional wall motion abnormalities Abnormal troponin
Vascular Toxicity	Presentation can vary widely from increased vasoreactivity to acute coronary syndrome and thus consult established guidelines
Hypertension	Established guidelines apply to cancer patients. Onset hypertension over 180 mmHg systolic or 110 mmHg diastolic should lead to the cessation of cancer therapeutics
Arrhythmias	Follow established guidelines

CTRCD: Cancer therapy related cardiac dysfunction; EF: Ejection Fraction; GLS: Global longitudinal Strain; MRI: Magnetic Resonance Imaging.

**Table 2:** Refs. [61,62,63]: primary categories of cardiac toxicities with common inciting cancer therapies.

Toxicity	Common Therapies
CTRCD	Anthracyclines, anti-HER2 therapies, TKIs, ICIs, CAR-T cell therapy, proteasome inhibitors, RAF/MEK inhibitors
Myocarditis	ICIs, anthracycline, fluoropyrimidines, and alkylating agents
Vascular Toxicities	GnRH agonists, androgen deprivation therapies, fluoropyrimidines, VEGF inhibitors, TKIs, CAR-T cell therapy, proteasome inhibitors, immunomodulatory drugs
Hypertension	VEGF inhibitors, Monoclonal antibody-base TKI, mTOR inhibitors, Proteasome inhibitors, anti-HER2 therapies, immunomodulatory drugs, RAF/MEK inhibitors
Arrhythmias	Alkylating agents, Anthracyclines, anti-metabolites, IL-2, Small molecule TKIs, Topoisomerase II inhibitors, Taxanes, ICIs, BRAF inhibitors, immunomodulatory drugs

CTRCD: Cancer therapy related cardiac dysfunction; HER2: Human epidermal factor Receptor 2; TKI: Tyrosine Kinase inhibitors; CAR-T: Chimeric antigen receptor T; ICI: Immune checkpoint inhibitor; GnRH: Gonadotropin releasing hormone; VEGF: Vascular endothelial growth factor; mTOR: mechanistic target of rapamycin.

**Table 3:** Modifiable and non-modifiable patient-related risk factors for CTR-CVT [62].

Modifiable Risk Factors	Non-Modifiable Risk Factors
Lifestyle risk factors ○ Obesity ○ Smoking ○ Physical activity ○ Poor diet ○ Excess Alcohol consumption Clinical Risk factors ○ Hypertension ○ Diabetes ○ Dyslipidemia	Clinical History ○ Previous or current CVDP ○ revious cardiotoxic treatments Age Genetics Sex

**Table 4:** Comparison of major professional society recommendations for cardiovascular risk assessment and surveillance in cardio-oncology.

Aspect	ESC 2022 (Cardio-Oncology Guidelines)	ASCO 2017/2023 Update (Prevention and Monitoring of CTRCD)	ICOS 2022–2023 (Consensus Statements)	ACC/AHA Recommendations * (Where Applicable)
Scope	Comprehensive guideline covering the entire cancer core continuum (prevention, surveillance, management, and survivorship) for all major cardiotoxic cancer therapies.	Focused on prevention and monitoring of cancer therapy-related cardiac dysfunction (CTCRCD) and heart failure in adult patients.	International consensus on definitions, classification, and multidisciplinary care in cardio-oncology.	General cardiovascular disease (CVD) prevention and management guidance applied to patients with cancer: not a dedicated cardio-oncology surveillance guideline.
Risk Assessment Framework	HFA/ICOS risk assessment tool (low, moderate, very high risk) based on patient-, disease-, and treatment-related factors.	Identifies increased-risk patients (age, prior CVD, CV risk factors, high risk therapies) but does not provide a formal risk scoring system.	Endnesses use of HFA/ICOS risk assessment tool; emphasizes standardized risk evaluation and multidisciplinary approach.	Uses traditional ASCVD risk assessment and clinical judgment to guide prevention and management.
Baseline Assessment Recommendations	History, physical exam, ECG, echocardiography (including LVEF and GLS), cardiac biomarkers (HP troponin) in patients at moderate, high, or very high risk.	Baseline echocardiogram reasonable in patients at increased risk for CTRCD; biomarkers may be considered in select high-risk patients.	Recommends baseline cardiovascular evaluation with history, ECG, imaging, and biomarkers in appropriate patients at risk.	Baseline evaluation guided by overall CVD risk; echocardiography not routinely recommended unless clinically indicated.
Surveillance During Therapy	Risk: and therapy-adapted surveillance with ECG, biomarkers, and echocardiography (including GLS) at defined intervals based on risk level and treatment status.	Echocardiogram and biomarkers le.g., troponin, NPI for patients receiving anthracyclines, HER2 therapy, and other high risk agents; frequency individualized.	Supports biomarker- and imaging-guided monitoring; frequency depends on risk and therapy, consistent with ESC framework.	No specific surveillance intervals for cancer therapies; manage cardiovascular complications and optimize CV risk factors.
Post-treatment/Survivorship Follow-up	Recommends long-term follow-up based on persistent risk, late cardiotoxicity potential, and survivorship care plans.	Survivorship recommendations provided for patients at risk of late CTRCD; less detailed intervals.	Emphasizes need for long-term care pathways and transition to survivorship programs.	Focuses on long-term CVD risk management and prevention in cancer survivors.
Biomarkers	Recommends use of NP (BNP/NT-proBNP) and troponin for risk assessment, monitoring, and early detection of cardiotoxicity.	Supports use of troponin and BNP/NT-proBNP for monitoring in patients receiving cardiotoxic therapies.	Endnesses biomarkers (troponin, NPI) for risk stratification and monitoring.	No specific recommendations for cardiac biomarkers in cancer therapy monitoring.
Imaging	Echocardiography with LVEF and GLS recommended; CMR or other imaging modalities in selected cases.	Echocardiogram is primary imaging modality; CMR may be considered in select situations.	Echocardiography (LVEF, GLS) recommended; CMR and other imaging tools in specific cases.	Echocardiography as clinically indicated; CMR for specific cardiovascular conditions.
Strengths	Most comprehensive, risk-adapted, and therapy-specific recommendations across all phases of cancer care.	Practical and widely endorsed guidance for prevention and monitoring of CTRCD.	Provides standardized definitions and global consensus to harmonize practice and research.	Strong evidence base for general CVD prevention and management.
Limitations	Many recommendations based on observational data or expert consensus; evidence gaps remain.	Narrower scope; less comprehensive for newer therapies and long-term surveillance.	Consensus-based; does not provide prescriptive surveillance schedules for all therapies.	Not designed for cardio-oncology; lacks detailed guidance on cancer therapy surveillance.

* ACC/AHA guidance refers to the 2019 ACC/AHA Guideline on the Primary Prevention of Cardiovascular Disease and the 2022 AHA/ACC/HFSA Guideline for the Management of Heart Failure, applied where relevant to patients with cancer. Abbreviations: ACC, American College of Cardiology; AHA, American Heart Association; ASCO, American Society of Clinical Oncology; CTRCD, cancer therapy-related cardiac dysfunction; CVD, cardiovascular disease; ECG, electrocardiography; ESC, European Society of Cardiology; GLS, global longitudinal strain; HFA, Heart Failure Association; HFSA, Heart Failure Society of America; ICOS, International Cardio-Oncology Society; NP, natriuretic peptide.

**Table 5:** Surveillance recommendations by risk category.

Risk Category	Clinical Profile	Surveillance Strategy (Imaging + Biomarkers)	Timing/Frequency	Escalation Triggers	Management Implications
Low Risk	No CV disease, low-risk therapy, normal baseline imaging/biomarkers	Baseline TTE + GLS; no routine serial imaging required unless symptomatic	No fixed interval; reassess only if clinical change	Symptoms or incidental abnormalities	Routine oncology follow-up Cardiology referral if CV toxicity develops (Class I)
Moderate Risk	CV risk factors or moderate-risk therapy exposure	TTE + GLS at baseline and peaking reassessment; consider biomarkers	Every 3–6 months depending on therapy type	GLS reduction (>15%), rising troponin/NP, or symptoms	Intensify surveillance; Cardiology referral if CV toxicity develops (Class I)
High Risk	Pre-existing CVD, high-risk therapy (anthracyclines, HER2, VEGF), or elevated baseline biomarker	Multimodal surveillance with TTE + GLS + serial biomarkers	Every 2–3 cycles or 3 months; earlier if abnormalities detected	GLS decline (>15%), biomarker elevation, early VVEF change	Early cardiology referral (Class I) Initiate cardioprotective therapy (Class IIa)
Very High Risk	Prior cardiotoxicity, multiple risk factors, high cumulative dose, or combination of cardiotoxic cardiotoxies	Early cardiology referral(class I), intensive monitoring with close integration of imaging and biomarker	Often every 1–2 cycles or ≤3 months, individualized based on trajectory	Any dynamic change (GLS, biomarkers, symptoms)	Proactive co-management; Risk/benefit discussion of cardiotoxic treatment (class I) or cardioprotective strategy (class IIa)

## Data Availability

No new data were created or analyzed in this study. Data sharing is not applicable to this article.

## Abbreviations

The following abbreviations are used in this manuscript:

CTR-CVT	cancer therapy-related cardiovascular toxicity
CTRCD	cancer therapy-related cardiac dysfunction
CVD	cardiovascular disease
GLS	global longitudinal strain
LVEF	left ventricular ejection fraction
ROS	reactive oxygen species
RNS	reactive nitrogen species
ICI	immune checkpoint inhibitor
CAR-T	chimeric antigen receptor T-cell therapy
VEGF	vascular endothelial growth factor
HER2	human epidermal growth factor receptor 2
TKI	tyrosine kinase inhibitor
HF	heart failure
TTE	transthoracic echocardiography
BNP	B-type natriuretic peptide
NT-proBNP	N-terminal pro-B-type natriuretic peptide
